# Novel epigenetic biomarkers for hematopoietic cancer found in twins

**DOI:** 10.2340/1651-226X.2024.40700

**Published:** 2024-09-18

**Authors:** Signe B. Clemmensen, Henrik Frederiksen, Jonas Mengel-From, Aino Heikkinen, Jaakko Kaprio, Jacob vB. Hjelmborg

**Affiliations:** a Department of Epidemiology, Biostatistics, and Biodemography, Institute of Public Health, University of Southern Denmark, Odense, Denmark; b Danish Twin Registry, Institute of Public Health, University of Southern Denmark, Odense, Denmark; c Department of Haematology, Odense University Hospital, Odense, Denmark; d Department of Clinical Research, University of Southern Denmark, Odense, Denmark; e Department of Clinical Genetics, Odense University Hospital, Odense, Denmark; f Institute for Molecular Medicine Finland FIMM, HiLIFE, University of Helsinki, Helsinki, Finland

**Keywords:** DNA methylation, EWAS, hematopoietic malignancy, survival analysis, twin study, heritability, FinnGen

## Abstract

**Background and purpose:**

This article aims to identify epigenetic markers and detect early development of hematopoietic malignancies through an epigenome wide association study of DNA methylation data.

**Materials and methods:**

This register-based study includes 1,085 Danish twins with 31 hematopoietic malignancies and methylation levels from 450,154 5’-C-phospate-G-3’ (CpG) sites. Associations between methylation levels and incidence of hematopoietic malignancy is studied through time-to-event regression. The matched case-cotwin design, where one twin has a malignancy and the cotwin does not, is applied to enhance control for unmeasured shared confounding and false discoveries. Predictive performance is validated in the independent Older Finnish Twin Cohort.

**Results and interpretation:**

We identified 67 epigenetic markers for hematopoietic malignancies of which 12 are linked to genes associated with hematologic malignancies. For some markers, we discovered a 2–3-fold relative risk difference for high versus low methylation. The identification of these 67 sites enabled the formation of a predictor demonstrating a cross-validated time-varying area under the curve (AUC) of 92% 3 years after individual blood sampling and persistent performance above 70% up to 6 years after blood sampling. This predictive performance was to a large extent recovered in the validation sample showing an overall Harrell’s C of 73%.

In conclusion, from a large population representative twin study on hematopoietic cancers, novel epigenetic markers were identified that may prove useful for early diagnosis.

## Introduction

Hematopoietic malignancies are cancers of the blood and blood-forming organs. They constitute 8–10% of all cancers diagnosed in the Nordic countries between 2017 and 2021 [1, 2]. In recent years, many contributions have been made to improve diagnostics and treatment. The ability to detect hematopoietic malignancy through blood samples instead of more invasive biopsies holds great promise to improve early diagnosis of patients. It is already used to some extent as blood cell type composition can provide information on abnormal levels which is seen in patients with leukaemia.

A recent study of hematologic malignancies in twins from four Nordic countries found that genetic influence on variation in cancer liability was moderate, which prompted a search for environmental factors [3]. Epigenetic studies can provide the link between environmental exposures and the effect they have on our genes. More specifically, epigenetic modifications governing gene expression, mainly DNA methylation and histone modification, have been found to play a central role in the understanding of tumour cell development [4]. For instance, a recent study found that DNA methylation is crucial for trans-differentiation of pre-B-acute lymphoblastic leukaemia cells into functional macrophages [5]. Another study proposed a model using peripheral blood DNA methylation profiles to predict future development of B-cell type non-Hodgkin lymphomas [6].

Multiple epigenetic determinants aiding in diagnosis classification and treatment have been found – an overview presented by Blecua et al. [7]. We seek to add to previous findings by exploiting the matched case-control twin design (also known as the cotwin control design), which further allows us to examine what governs these epigenetic dysregulations. The matched design where one twin has a malignancy and the cotwin does not ensures control for unmeasured shared confounding, that is, shared genes and shared environment (e.g., environmental exposures during childhood). Monozygotic (MZ) twins are genetically identical at the sequence level, while dizygotic (DZ) twins share on average 50% of their segregating genetic material [8]. A within-pair difference in outcome statistically reflected in difference in exposure between twin and cotwin being MZ would indicate that non-shared environmental factors govern the association [9]. Limitations of the design are explained in the methods section.

In this study, we performed an epigenome wide association study (EWAS) of hematopoietic malignancy in Danish twins aiming to utilise the matched case-control twin design to identify possible epigenetic DNA methylation markers and to describe the influence of genetic and environmental exposures. We further aimed to qualify methylation markers aiding in the prediction of development of these malignancies.

## Materials and methods

### Study population

This EWAS analyses methylation data derived from venous blood samples taken from 1,013 MZ and 72 DZ individual twins including 505 MZ and 36 DZ complete pairs. Data were collected by the Danish Twin Registry through three studies: the Longitudinal Study of Aging Danish Twins (LSADT) [10], the Middle Aged Danish Twins Study (MADT) [11] and a study of extremely birth weight-discordant twins (BWD) [12] (See Table 1). Information on birthdate and life status was obtained via linkage to the Danish Civil Registration System.

**Table 1:** Characteristics of the study population.

Cohort	LSADT	MADT	BWD	Total
*N* individual twins	310	491	284	1,085
*N* (%) female twins	214 (69)	225 (46)	136 (48)	575 (53)
*N* (%) monozygotic twins	238 (77)	491 (100)	284 (100)	1,013 (93)
Year of blood sampling	1996–1997	2008–2011	2009–2010	
Median age at blood sampling (IQR), years	77.1 (75.6–80.4)	66.0 (61.3–71.2)	57.7 (33.9–63.8)	
*N* incident hematopoietic malignancies^a^	12	16	3	31
Median age at diagnosis	87.2	72.7	74.5	

a Incident malignancies diagnosed after individual blood sampling. In case of multiple diagnoses per individual (rare in this study), time to first diagnosis was used.

LSADT: Longitudinal Study of Aging Danish Twins Study; MADT: Middle Aged Danish Twins Study; BWD: birth weight-discordant twins; IQR: Interquartile range.

Cancer data were provided from the National Cancer Register with follow-up from 1943 to 2021. Cancer diagnoses were based on the International Statistical Classification of Diseases and Related Health Problems, Tenth Revision (ICD-10) and were grouped according to the NORDCAN classification system [2]. When identifying lymphoma incident cases, the International Classification of Diseases for Oncology, Third Edition (ICD-O-3) was used along with ICD-10. This study includes the following hematopoietic malignancies: Hodgkin lymphoma (C81), non-Hodgkin lymphoma (C82–86), multiple myeloma (C90) and leukaemia (C91–95) as well as myeloproliferative diseases (D45 + D47) and myelodysplastic syndromes (D46). The incidence of cancer among twins has been demonstrated to mirror that of the general population [3, 13].

### DNA methylation data

DNA methylation data were obtained for 485,512 CpG sites using the Infinium 450K HumanMethylation BeadChip. Twin pairs were analysed on the same array. The three data sets, LSADT, MADT and BWD, were analysed on different occasions as explained in Svane et al. [14]. Data preprocessing, quality control and normalisation was done using the R packages MethylAid [15] and minfi [16]. Further details on the process including requirements made in quality assessment are provided by Soerensen et al. [17].

A total of 450,154 overlapping CpG sites were included in this study.

Blood cell type composition (monocytes, lymphocytes and eosinophils) was available for most of the MADT and BWD twins. For the remaining individuals, including LSADT, proportions of blood cell types were imputed as described by Debrabant et al. [18].

Before statistical analysis, the methylation beta-values were corrected for cell type composition and batch effects using a linear mixed model with sex, age at entry, zygosity, data set and cell type composition as fixed effects and sample plate and position as random effects similar to Svane et al. [14]. Study entry was defined by time of blood sampling. The resulting residuals, standardised to a standard deviation of one, were used in the statistical analyses.

### Validation in Finnish twins

For external validation, we used data from 436 twins (349 females) from the Older Finnish Twin Cohort [19, 20]. Among these twins were seven incident hematopoietic malignancies. Median age at blood sampling was 65 years (IQR [Interquartile range]: 62–69 years) and median follow-up time was 11 years (IQR: 7–20 years) with end of follow-up in 2018. DNA methylation data stem from blood samples and was generated on the 450K and EPIC platforms as described in [19]. Sample quality control and normalisation of data were done using the R package meffil [21] as described in Heikkinen et al. [22]. The methylation *b*-values were corrected for cell type composition using linear regression.

### Statistical analysis

We performed an epigenome-wide association analysis to find CpG sites with a significant association to time to development of hematopoietic malignancy followed by development of a prediction model. Malignancies diagnosed before time of blood sampling were considered as non-cases. The methods applied for prediction modelling and validation are similar to what was done by Svane et al. [14]. All analyses were performed using the statistical software *R* version 4.1.0 [23].

### Epigenome-wide association analysis

The association between methylation level of individual CpG sites and time to development of hematopoietic malignancy was analysed using robust variance estimation to account for twin pair-specific similarities. Two approaches were applied: Cox frailty and Fine-Gray regression. Censoring at end of follow-up in 2021 and competing risk of death was considered. The Cox frailty model produces hazard ratios (HR) and Fine-Gray gives sub-distribution HR. The *R* packages survival [24] and mets [25, 26] were used for the analyses.

All results were adjusted for multiple testing using the Benjamini-Hochberg procedure. CpG sites with false discovery rate (FDR) < 0.05 were compared to findings in the literature related to hematopoietic malignancy. Because of sensitivity in model fit, significant CpG sites were identified as those (further) having unadjusted *p*-values <1e-7 on both scales: hazard and risk. The Cox proportional hazards assumption was assessed for the identified CpG sites using scaled Schoenfeld residuals. Phenotypes associated (*p* < 1e-5) with variants of genes related to the significant CpG sites, were looked up in FinnGen data freeze 10 (*n* = 412,181 individuals) from December 2023 [27].

Average causal exposure effect ratios (ATE risk ratios) were estimated 5 years after blood sampling for the identified CpG sites using G-estimation under standard causal assumptions on the Fine-Gray model described previously [28]. They were used for comparison of risk of hematopoietic malignancy among those with high versus low methylation levels defined by dichotomisation of standardised residuals using zero as threshold.

The identified CpG sites were further assessed (1) in a matched analysis using a stratified Cox model with twin pair specific baseline hazards to control for unobserved, shared confounding, and (2) by estimating the intraclass correlation (ICC) in methylation level within MZ and DZ twin pairs. Noteworthy, the ICC in MZ pairs provides an upper bound for the heritability when assuming the biometric polygenic variance components model (the ADCE model) from quantitative genetics [25]. While the matched case-control twin design, in general, will control for unmeasured shared confounding, severe bias may be induced by non-shared confounders less similar within the pair. Therefore, the ordinary individual regression needs to be applied in parallel [29, 30].

For validation of each of the identified CpG sites, we computed a five-fold cross-validated Harrell’s C for a Cox model with sex, zygosity, age at blood sampling and CpG methylation level as covariates and compared to a Cox model without the latter.

### Prediction modelling and validation

To identify candidates for a predictor of hematopoietic malignancy, we applied the least absolute shrinkage and selection operator (LASSO), from the R package glmnet [31], in a Cox model on the CpG sites with unadjusted *p*-values <1e-7 in both models in the EWAS. Sex and age at blood sampling were forced to be included as covariates. There were up to 2.7% missing values in the CpG sites. Through imputation, each missing value was replaced by its expected value in a linear regression of sex, age at blood sampling, zygosity, data set and cell type composition (monocytes, lymphocytes and eosinophils). Imputation was done on the raw methylation values and the output adjusted through a mixed linear model as stated previously to obtain the residuals used in the prediction model.

We applied stability selection and ran 1,000 replications of the LASSO on randomly selected subsets containing half of the sample and chose the smoothness parameter to be the sum of the minimizer of the partial likelihood under 10-fold cross-validation and half of the standard error [14]. CpG sites chosen in more than 80% of the replications were included in the prediction model.

For validation of each of the CpG sites selected for the predictor, we computed a five-fold cross-validated Harrell’s C for a Cox model with sex, zygosity, age at blood sampling and CpG methylation level as covariates and compared to a Cox model without the latter. Additionally, we computed an overall five-fold cross-validated Harrell’s C for a Cox model with sex, age at blood sampling and all predictor CpG sites as covariates and compared to a Cox model with only sex and age at blood sampling. The predictive ability over time of a model with beforementioned covariates was estimated using the R package timeROC [32] as a five-fold cross-validated, time-varying AUC.

To assess whether the effect of the predictor was independent of underlying genetic effects, we did a matched twin pair analysis of the MZ twins using a stratified Cox model on the predictor CpG sites with no other covariates. A correlation plot was made to illustrate pairwise associations between the predictor CpG sites. As sensitivity analysis, we assessed the influence of outliers among the identified predictors by excluding these individuals.

### Validation in Finnish twins

The CpG sites identified as significant in both modelling approaches in the individual EWAS were validated in the Finnish data by computing Harrell’s C for a Cox model with each of the identified CpGs as covariate adjusting for sex and age. This was compared to Harrell’s C for a basic model with sex and age only. Similar validation was done for each of the sites identified for the predictor. To validate the combined predictor, an overall Harrell’s C for a Cox model with all the predictor CpGs along with sex and age was estimated. We further attempted to assess the predicted ability over time using time-varying AUC as described previously.

## Results

We analysed time-to-event data of methylation levels of 450,154 CpG sites measured in 1,085 individual twins, of whom 26 MZ and 5 DZ twins were diagnosed with a hematopoietic malignancy (Table 1). The median time from blood sampling to diagnosis was 6.3 years with IQR 4.4–10.3 years.

### Epigenome-wide association analysis and validation

Using Cox frailty and Fine-Gray regression, we conducted an epigenome-wide association study of association between methylation levels and incidence of hematopoietic malignancy (Manhattan plots provided in Supplementary Figures 1 and 2). We identified 15,432 CpG sites with an FDR < 0.05 out of which 71 overlapping sites had an association *p*-value below 1e-7. The Cox proportional hazards assumption was verified for 67 out of these 71 identified CpG sites. Through the FinnGen study, we found 12 of these 67 sites linked to genes that were associated with hematologic malignancies and further 16 sites were linked to genes associated to other immune disorders or diseases of the blood (Supplementary Table 4). Association estimates from the two regression models were very similar and results from the Fine-Gray model are provided for beforementioned 12 sites in Table 2, while the Cox estimates are provided in Supplementary Table 1. The HR for 10 of these sites point in the direction of hypomethylation being associated with increased risk of hematopoietic malignancy. The remaining two HR point in the opposite direction. A full list of the results from the 15,432 CpG sites are provided in Supplementary Table 5.

**Table 2:** Estimates from the Fine-Gray model for the 12 CpG sites with *p* < 1e-7 in the epigenome-wide association analysis that were associated with hematologic malignancy in FinnGen.

CpG Site	sHR	*p*	FDR	ATE ratio	*p*
cg00461770	0.69	6.22e-16	1.85e-13	0.44	7.77e-04
cg00984696	0.58	5.25e-08	4.93e-06	0.32	6.97e-07
cg02383130	1.31	1.29e-18	6.00e-16	1.22	0.623
cg03140889	0.67	6.08e-09	6.48e-07	1.12	0.765
cg04335343	0.66	7.90e-11	1.11e-08	0.29	8.99e-10
cg04387984	0.62	5.56e-09	5.98e-07	0.40	1.19e-04
cg04504627	0.66	1.70e-18	7.74e-16	0.29	2.30e-09
cg05575733	0.68	1.46e-12	2.62e-10	0.42	3.79e-04
cg08111284	0.68	2.39e-13	4.78e-11	0.54	0.0171
cg09551472	1.60	2.57e-12	4.46e-10	2.25	0.134
cg21609584	0.60	4.98e-11	7.17e-09	0.42	2.81e-04
cg26074430	0.75	1.11e-18	5.20e-16	0.43	5.48e-04

sHR: sub-distribution hazard ratio; ATE ratio: average causal CpG effect ratio.

The matched twin pair analysis yielded HR pointing in the same direction as beforementioned individual level analysis, but only 2 out of 12 were statistically significant (Supplementary Table 2). This provides rather strong support of the findings in a setting controlling for unobserved, shared confounding. The matched twin pair analysis indicates that the association between CpG methylation levels and hematopoietic malignancy depends little on genetic effects and points toward environmental exposures, although the FinnGen GWAS findings indicate there are also contribution of underlying genetic variants in these 12 genes. The average causal CpG effect comparing risk of hematopoietic malignancy among those with high versus low methylation levels are provided in Table 2. For nine of the sites, the significant ratios indicate that the risk of hematopoietic malignancy 5 years after blood sampling is about 2–3 times higher among individuals with low methylation levels.

Within-pair correlations in methylation level by zygosity are listed in Table 3 along with chromosome, gene name, region and an overall grouping of associated hematologic malignancy in FinnGen. The MZ correlations are significantly different from zero for seven sites and the highest correlation value suggest a moderate upper bound of genetic influence on each of the CpG sites related to hematopoietic malignancy at 0.34. Only one of the DZ correlations is different from zero, and the values generally have wide 95% Confidence Interval (CI), reflecting small sample size. It is worth noting that half of the validated CpG sites in Table 3 have MZ within pair correlations above 0.15 allowing for a potential moderate heritability of the methylation pattern related to hematopoietic malignancy.

**Table 3:** The 12 CpG sites with *p* < 1e-7 in the epigenome-wide association analysis that were associated with hematologic malignancy in FinnGen.

CpG Site	MZ Correlation	DZ Correlation	Chr	Gene	Region	FinnGen ass. phenotype^a^
cg00461770	0.04 (−0.04 – 0.13)	−0.08 (−0.76 – 0.68)	1	DFFB	South shore	leukaemia
cg00984696	0.27 (0.19 – 0.35)	−0.41 (−0.71 – 0.02)	3	KALRN	Open sea	NHL
cg02383130	0.03 (−0.06 – 0.12)	0.08 (−0.18 – 0.34)	7	AMPH	South shelf	leukaemia and NHL
cg03140889	0.09 (0 – 0.18)	0.18 (−0.14 – 0.46)	8	SULF1	Open sea	leukaemia and NHL
cg04335343	0.15 (0.06 – 0.24)	0.09 (−0.14 – 0.32)	6	GABBR1	South shore	leukaemia and NHL
cg04387984	0.16 (0.07 – 0.24)	0.21 (−0.18 – 0.55)	3	LPP	Open sea	NHL
cg04504627	−0.05 (−0.13 – 0.04)	0.23 (−0.27 – 0.63)	6	HLA-DRA	Open sea	leukaemia, HL and NHL
cg05575733	0.34 (0.26 – 0.41)	0.31 (−0.25 – 0.72)	17	NXN	Island	NHL
cg08111284	0.15 (0.06 – 0.23)	0.64 (0.41 – 0.8)	6	TNXB	South shore	HL and NHL
cg09551472	0.14 (0.05 – 0.22)	0.25 (−0.09 – 0.54)	6	POM121L2	Island	NHL
cg21609584	0.30 (0.21 – 0.37)	−0.07 (−0.36 – 0.23)	2	NPAS2	Open sea	Multiple myeloma
cg26074430	−0.03 (−0.12 – 0.06)	0.37 (−0.03 – 0.67)	6	RXRB	North shelf	NHL

Within pair correlation in MZ and DZ twin pairs.

MZ: monozygotic; DZ: dizygotic; Chr: Chromosome; NHL: non-Hodgkin lymphoma; HL: Hodgkin lymphoma.

a Gene variant has been associated with phenotype (*p* < 1e-5) related to hematologic malignancy in FinnGen. Categorized phenotypes are listed here. Further details provided in Supplementary Table 4.

**Figure 1:**
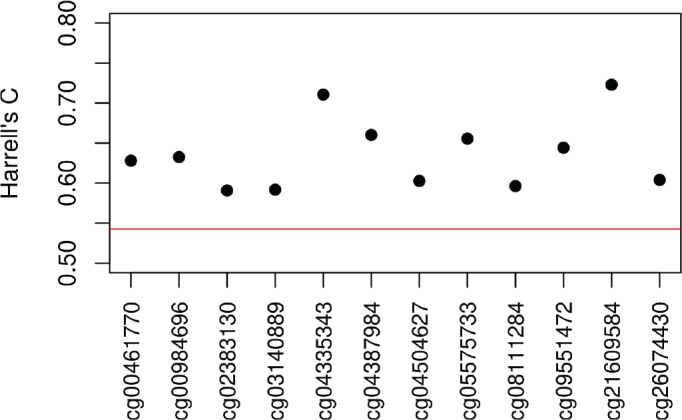
Five-fold cross-validated Harrell’s C for the 12 identified CpG sites for a Cox model with sex, zygosity, age at blood sampling, and CpG methylation level as covariates. The red reference line is for comparison with Harrell’s C for a basic model without CpG methylation levels.

Figure 1 shows five-fold cross-validated Harrell’s C for each of the identified CpG sites in the range 0.58–0.73 which suggests improvement of the model when including the CpG methylation levels compared to the basic model with a Harrell’s C of 0.54.

### Prediction modelling and validation

We identified 12 candidates for a predictor of hematopoietic malignancy. For each of these sites, characterisation in terms of HR, within pair MZ and DZ correlations and location are listed in Table 4. The MZ correlations are significantly different from zero for eight sites and the highest correlation value suggest a moderate upper bound of genetic influence on each of the CpG sites related to hematopoietic malignancy at 0.39.

**Table 4:** The 12 CpG sites selected as predictors of hematopoietic malignancy along with sex and age at blood sampling.

Covariate	HR	SE	*p*	MZ Cor	DZ Cor	Chr	Gene name	Region
cg00417408	1.46	0.094	<0.001	0.10 (0.02 – 0.18)	0.36 (−0.16 – 0.73)	7	TMEM176A; TMEM176B	Island
cg01671681	0.74	0.149	0.042	0.29 (0.2 – 0.36)	0.26 (−0.03 – 0.51)	3	PLCH1	Island
cg02383130	1.01	0.115	0.93	0.03 (−0.06 – 0.12)	0.08 (−0.18 – 0.34)	7	AMPH	South shelf
cg08512702	1.23	0.117	0.072	0.08 (−0.01 – 0.17)	−0.01 (−0.23 – 0.2)	4	LOC285548	Island
cg08549335	0.57	0.182	0.002	0.26 (0.17 – 0.34)	0.15 (−0.23 – 0.48)	7	ZNRF2	Open sea
cg13280788	1.00	0.185	0.98	0.38 (0.31 – 0.46)	0.04 (−0.23 – 0.31)	17	HOXB3	North shore
cg17931529	1.00	0.117	0.99	0.31 (0.24 – 0.39)	0.53 (0.23 – 0.74)	19	MOBKL2A	South shore
cg22759800	0.80	0.143	0.12	−0.03 (−0.12 – 0.05)	0.48 (0.09 – 0.74)	15	-	Island
cg23112821	0.75	0.167	0.093	0.31 (0.23 – 0.39)	−0.12 (−0.35 – 0.13)	14	-	North shore
cg23816347	1.34	0.156	0.058	0.02 (−0.06 – 0.11)	0.28 (−0.04 – 0.55)	3	CCDC37	Island
cg23869158	0.86	0.155	0.32	0.39 (0.32 – 0.46)	0.05 (−0.23 – 0.33)	13	LRCH1	North shore
cg24890104	0.87	0.195	0.48	0.38 (0.31 – 0.45)	−0.25 (−0.68 – 0.3)	6	-	South shore
Sex (female)	0.63	0.422	0.27					
Age at blood sampling (years)	1.03	0.022	0.12					

HR: hazard ratio; SE: standard error; MZ: monozygotic; DZ: dizygotic; Cor: Correlation; Chr: Chromosome.

**Figure 2:**
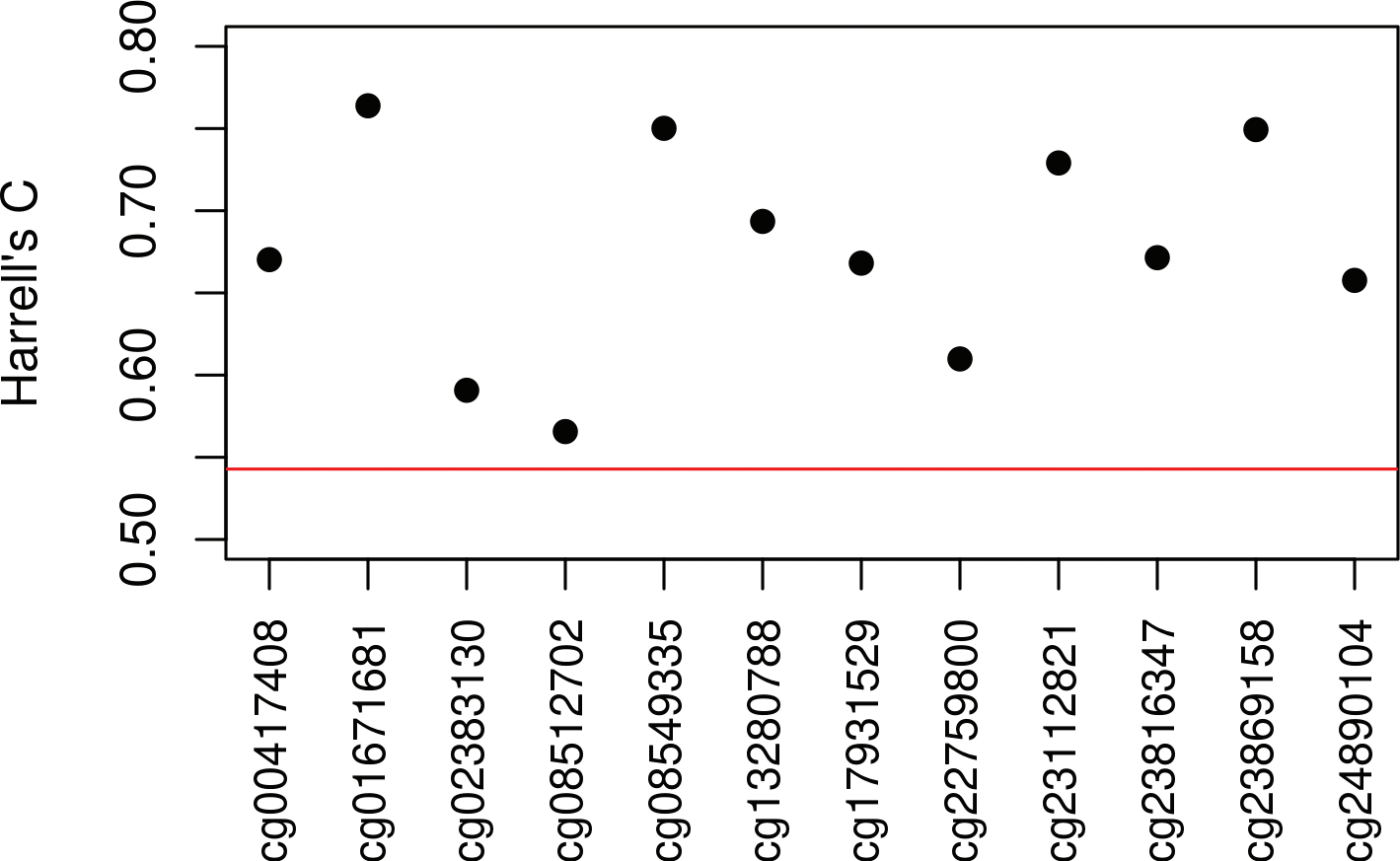
Five-fold cross-validated Harrell’s C for the 12 predictor CpG sites for a Cox model with sex, age at blood sampling, and CpG methylation level as covariates. The red reference line is for comparison with Harrell’s C for a basic model without CpG methylation levels.

Figure 2 shows five-fold cross-validated Harrell’s C for each of the predictor CpG sites in the range 0.57–0.77 which suggests improvement of the model when including the CpG methylation levels compared to the basic model with a Harrell’s C of 0.54. A similar pattern was seen for an overall Harrell’s C of 0.85 for a model with all predictor CpG sites, sex, and age at blood sampling.

**Figure 3:**
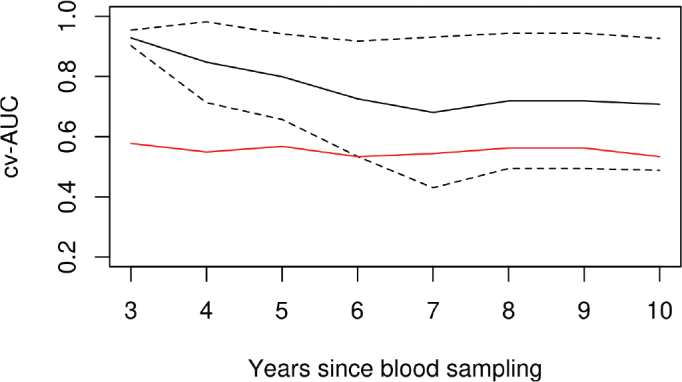
Five-fold cross-validated time-varying AUC (cv-AUC) with 95% CI for a prediction model of hematopoietic malignancy with sex, age at blood sampling, and CpG methylation level as covariates. The red reference line is for comparison to a basic model without CpG methylation levels.

The cross-validated, time-varying AUCs for the model with all 12 sites selected for the predictor along with sex and age at blood sampling and the model with only the two latter covariates are shown in Figure 3. The prediction performance shows the ability of 92% 3 years after blood sampling and persistent performance above 70% up to 6 years after blood sampling.

The pairwise correlations between the predictor CpG sites are illustrated in Supplementary Figure 3. They indicate a rather low co-dependence among the CpG sites chosen for the predictor. The sensitivity analysis excluding outliers resulted in slightly higher correlations, but very similar regression estimates and predictive abilities. A matched twin pair analysis of the MZ twins using the predictor CpG sites with no other covariates resulted in a *p*-value of 0.07.

### Validation in Finnish twins

Among the reported CpG sites shown in Figure 1, 11 out of 12 were available for replication in the Finnish cohort (Supplementary Figure 4). Despite having only seven cases available in the Finnish cohort, a significant (*p* = 0.003) recovery of cg21609584 was detected in association with hematopoietic malignancy (HR = 0.32) (Supplementary Table 3). Harrell’s C for half of the sites were above 55% and especially two sites had very high values (above 70%): cg02383130 and cg09551472. The CpG sites’ combined predictive ability using the 10 out of 12 sites represented in the Finnish cohort was high (Harrell’s C: 73%) even with two sites missing, but the predictive performance over time (see Figure 3) could not be validated because of missing values. Promising values of site-specific Harrell’s C of larger than 70% were seen for cg01671681 and cg08549335, the latter at 85% (Supplementary Figure 5).

## Discussion

Our main finding of epigenetic methylation sites associated with hematopoietic malignancies contributes to understanding the disease aetiology. We have identified 67 epigenetic markers of which 12 are linked to genes associated with hematologic malignancy, as seen in FinnGen. For instance, cg00984696 and cg05575733 which are both related to non-Hodgkin lymphoma (NHL) and have MZ within pair correlations allowing for potentially moderate genetic influence. The remaining 55 CpG sites are left for further investigation. We have estimated average causal CpG effects comparing risk of hematopoietic malignancy 5 years after individual blood sampling among those with high versus low methylation levels using the Fine-Gray model. To the best of our knowledge, this has not been pursued previously. Moreover, the established prognostic index of methylation markers for disease risk discrimination performs persistently at a high level up to 6 years after blood sampling. This could prove to be helpful in improving early disease identification through epigenetic markers and it demonstrates the importance of epigenetic information for a deeper understanding of these malignancies for which external environmental determinants are known to be influential [33, 34]. The matched twin pair analysis of MZ twins and effective controlling for genetic similarity, indicate that the predictor was mostly independent of underlying genetic effects and that we should look for environmental influences.

Dysregulation in DNMT3A/B, TET1/2 and IDH1/2 has been observed repeatedly among individuals diagnosed with hematopoietic malignancies [35]. None of the 67 CpG sites identified in this study were linked to these genes. However, the following CpG sites were among the 15,432 sites with FDR < 0.05 in the EWAS: cg25456062 (DNMT3A), cg31352228 (DNMT3B), cg31351813 (DNMT3B), and cg90627448 (IDH2). Another study applied machine learning methods to form a prediction model aiming to aid early detection of B-cell NHL. They focus on polycomb, HOX and PAX5 genes as hypermethylation was found to be associated with future development of NHL [6]. Again, none of the 67 CpG sites identified in this study were linked to these genes. However, HOXB3, a gene associated with acute myeloid leukaemia (AML), was among the 15,432 sites with FDR < 0.05. Similar means of epigenome wide methylation profiling and application of machine learning has been used to identify 20 markers of ALL and 23 markers of AML [36]. Surprisingly, none of the identified CpGs could be validated in our findings. It may be because of the difference in disease outcome – the present study aimed broader by considering all hematopoietic malignancies combined.

Most studies focus on specific subtypes of hematopoietic malignancies and are thus able to provide markers that are expectedly better suited for diagnostics. However, evidence for shared etiological factors across subtypes of hematopoietic malignancies were assessed in a Swedish study of more than 150,000 patients [37]. Besides, studying hematopoietic malignancies combined is potentially more powerful in terms of detection of DNA methylation aberrations arising earlier in the process of blood cell formation, that is, at the top of the hematopoietic hierarchy where hematopoietic stem cells have yet to be restricted to a certain lineage [4].

The matched case-control twin design provides natural confounder control not otherwise obtainable for the scenario in which non-genetic effects are present for the association of methylation sites with hematopoietic malignancies. Identifying these will have importance for the prevention. We note, that the genetic variation accounted for through matching is primarily germline. However, if causal somatic variants provide CpG changes, the matched design would in theory still be of high validity and useful for the identification. Other strengths of the study are substantial follow-up time, the use of population-wide registers for information on diagnosis and time of death, and the representativity of two of the cohorts (MADT and in LSADT) where twins from certain birth cohorts were invited at random. The third cohort is not entirely random as twins were selected based on differing birthweights. However, this is not expected to influence the association between DNA methylation and risk of hematopoietic malignancy.

The overlap between the CpG sites linked to genes associated with haematological malignancy in FinnGen and those sites selected for the predictor was very small – only one CpG site was present in both. This could be an indication that a more extensive search for linked genes among the 55 ‘unlinked’ CpGs would prove fruitful.

Our reported findings of associated and predicted CpG sites were validated to a satisfactory extent in the independent and population representative Finnish twin sample. In particular, two CpG sites were directly related to leukaemia (cg02383130) and NHL (cg02383130 and cg09551472). There is some uncertainty in these validation estimates as seen from the Harrell’s C being lower than 0.5 for the baseline model and some of the CpG sites, that is the prediction being worse than chance. This is expectedly a result of the limited number of events in the Finnish cohort.

Adjusting for cell type composition may on one hand account for important unmeasured confounding (closing a backdoor path), but on the other hand induce colliding effects if altered cell type composition is caused by the methylation process and the disease in question. We decided on the first option and expect the latter to be of limited influence.

A weakness in our study is that the choice of time scale does not incorporate malignancies diagnosed before individual blood sampling. In the data set, eight individuals were diagnosed with a hematopoietic malignancy 1–9 years prior to blood sampling. There is potential for further studies in this regard, for example assessing methylation levels before and after diagnosis and, further, the differences within twin pairs. Another limitation is lack of measurements of specific environmental exposures. Smoking has been shown to have a strong effect on methylation and smoking discordant pairs have lymphoma-associated biomarker differences [38]. Hence, our findings could be explained by smoking behaviour as is the case for many cancers. However, as some of the findings relate to known hematopoietic malignancy linked genes, smoking may be a moderator of their effects. This could be a topic for further investigation.

In a follow-up, one could extend analysis of the 4 CpG sites failing the proportional hazards assumption, for example by allowing for time-varying effects. Alternative approaches may also be of interest for the CpG sites complying with the proportional hazards assumption since ‘not rejecting’ could be because of the low number of events. For instance, one could pursue the MZ within pair dependence in CpG level as a selection criterion for the CpG sites to be considered for the prediction model since high dependency presumably reflects underlying genetic control and importance.

Our present study on epigenetic findings may in combination with a previous study on genetic and environmental influence on hematologic malignancies in Nordic twins [3] provide the perspective that environmental factors interact with genetic effects, having epigenetic mechanisms as a moderator. An example of this is breast implant-associated anaplastic large cell lymphoma for which a recent study demonstrated recurrent mutations in epigenetic regulators in addition to alterations of genes in the JAK-STAT pathway [39]. In view of this, we conjecture that tattoo ink deposits in the lymph system could be one such environmental exposure having the cause of altered epigenetic states influencing genetically regulated cell-proliferation which initiates cancerous states. This gene by tattoo environmental exposure will be pursued in later studies to come with established twin cohort data.

The finding that DNA methylation plays an important role for the risk of hematopoietic malignancies, in particularly conveying environmental exposures, opens the perspective of identifying preventive measures. As a perspective, we will follow up on this considering environmental candidates of association reported from other studies [7, 35] in a study to come.

In conclusion, we have identified novel methylation sites associated to risk of hematopoietic malignancy. Further, the finding that non-genetic effects take part in the abnormal cell proliferation points towards preventive potentials to be discovered.

## Supplementary Material

Novel epigenetic biomarkers for hematopoietic cancer found in twins

Novel epigenetic biomarkers for hematopoietic cancer found in twins

Novel epigenetic biomarkers for hematopoietic cancer found in twins

## Data Availability

Restrictions apply to the availability of these data. Requests to access these data need to be made to the Danish Twin Registry.
