# Supplementary material for: Novel epigenetic biomarkers for hematopoietic cancer found in twins
Source: Acta Oncol. 2024 Sep 18;63:40700. doi: 10.2340/1651-226X.2024.40700 (PMC11423697; doi:10.2340/1651-226X.2024.40700)

Supplementary material has been published as submitted. It has not been copyedited, or typeset by Acta Oncologica

Supplementary Figure 1. Manhattan plot for epigenome-wide association analysis using the Cox frailty model. The horizontal line represents the significance level 1e-7.

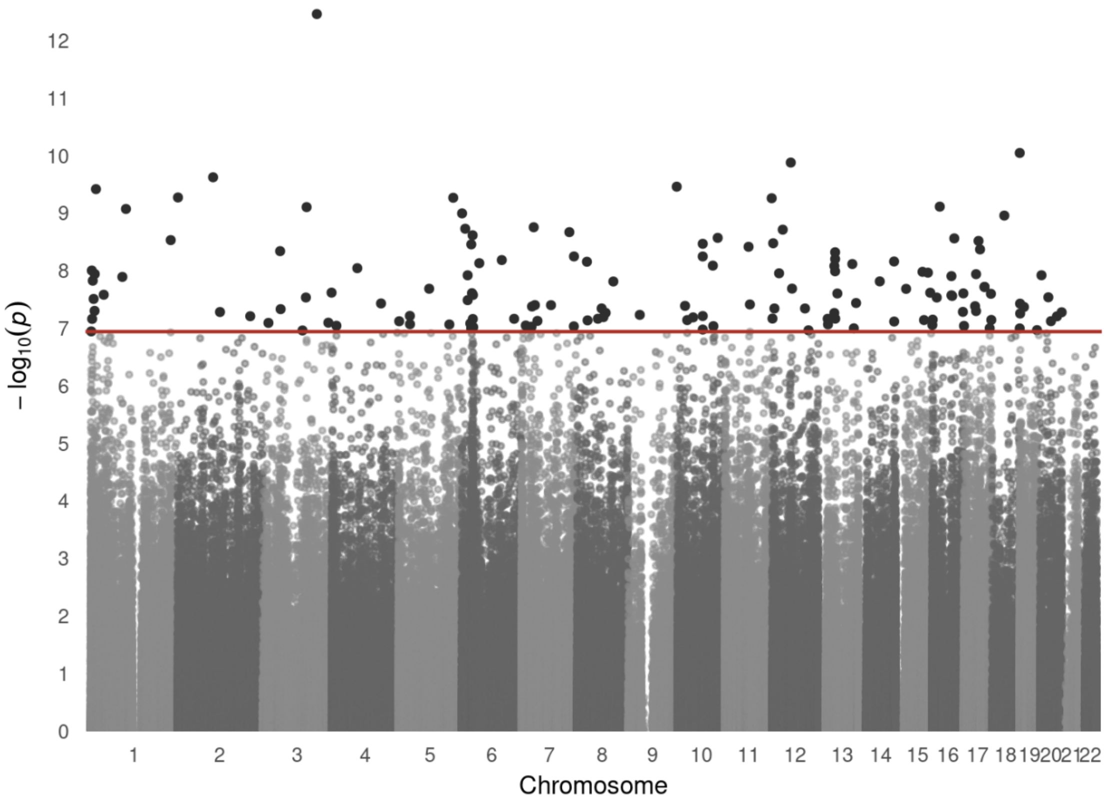

Supplementary Figure 2. Manhattan plot for epigenome-wide association analysis using the Fine-Gray model. The horizontal line represents the significance level 1e-7.

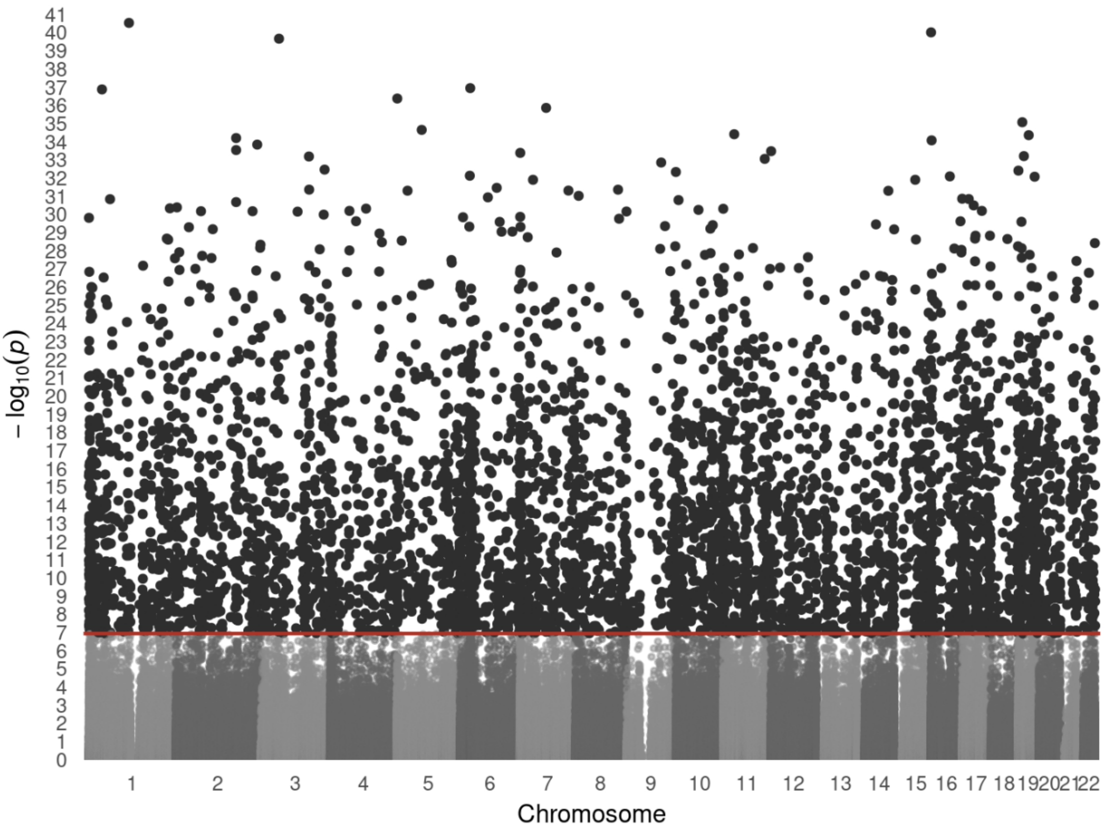

Supplementary Table 1. Regression coefficients from the Cox frailty model for the 12 CpG sites with p<1e-7 in the epigenome-wide association analysis that were associated with hematologic malignancy in FinnGen.

CpG Site	HR	p	FDR
cg00461770	0.70	9.86e-09	1.06e-04
cg00984696	0.58	2.90e-08	1.79e-04
cg02383130	1.31	3.93e-08	2.19e-04
cg03140889	0.67	4.49e-08	2.30e-04
cg04335343	0.61	2.44e-08	1.70e-04
cg04387984	0.62	7.94e-08	2.95e-04
cg04504627	0.67	2.62e-08	1.73e-04
cg05575733	0.68	9.03e-08	3.13e-04
cg08111284	0.69	6.85e-08	2.78e-04
cg09551472	1.58	9.93e-08	3.26e-04
cg21609584	0.57	2.36e-10	2.66e-05
cg26074430	0.76	9.50e-08	3.19e-04

Supplementary Table 2. Regression coefficients from the matched analysis.

CpG Site	HR	SE	p
cg00461770	0.34	0.49	0.026
cg00984696	0.76	0.24	0.26
cg02383130	1.16	0.17	0.38
cg03140889	0.85	0.16	0.33
cg04335343	0.66	0.27	0.12
cg04387984	0.68	0.25	0.13
cg04504627	0.60	0.30	0.085
cg05575733	0.29	0.60	0.037
cg08111284	0.76	0.27	0.30
cg09551472	1.33	0.27	0.30
cg21609584	0.59	0.31	0.087
cg26074430	0.59	0.32	0.11

Supplementary Figure 3. Correlations of the 12 CpG sites selected for the prediction model, n=1,085.

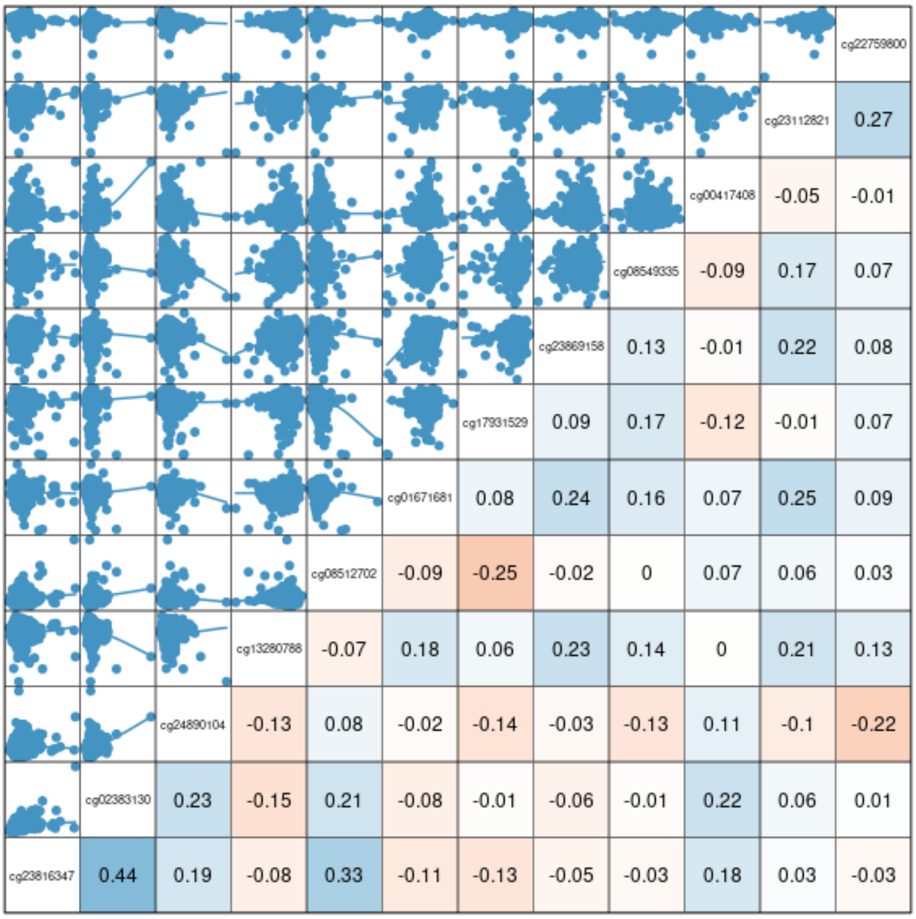

Supplementary Figure 4. Replication in Finnish cohort. Harrell's C for the 11 of the identified CpG sites for a Cox model with sex, zygosity, age at blood sampling, and CpG methylation level as covariates. The red reference line is for comparison with Harrell's C for a basic model without CpG methylation levels.

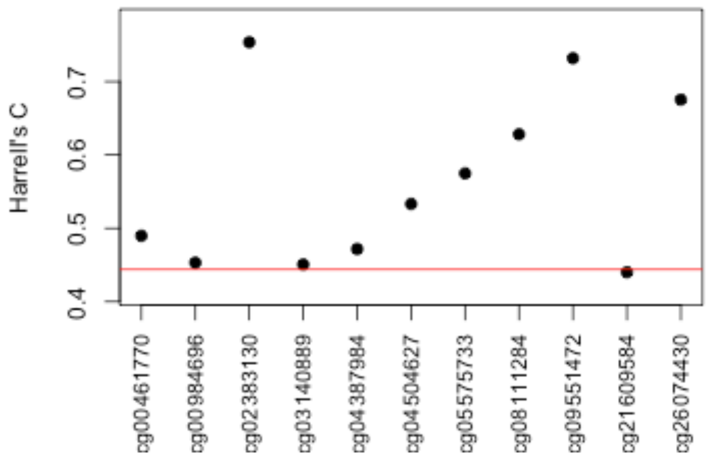

Supplementary Table 3. Replication in Finnish cohort. Hazard ratios for the 11 of the identified CpG sites for a Cox model with sex, zygosity, age at blood sampling, and CpG methylation level as covariates.

CpG Site	HR	p
cg00461770	0.84	0.61
cg00984696	1.06	0.85
cg02383130	1.53	0.32
cg03140889	7.58	0.34
cg04387984	1.82	0.33
cg04504627	1.05	0.90
cg05575733	1.39	0.64
cg08111284	0.99	0.98
cg09551472	0.97	0.94
cg21609584	0.32	0.003
cg26074430	1.72	0.18

Supplementary Figure 5. Replication in Finnish cohort. Harrell's C for the 10 of the predictor CpG sites for a Cox model with sex, age at blood sampling, and CpG methylation level as covariates. The red reference line is for comparison with Harrell's C for a basic model without CpG methylation levels.

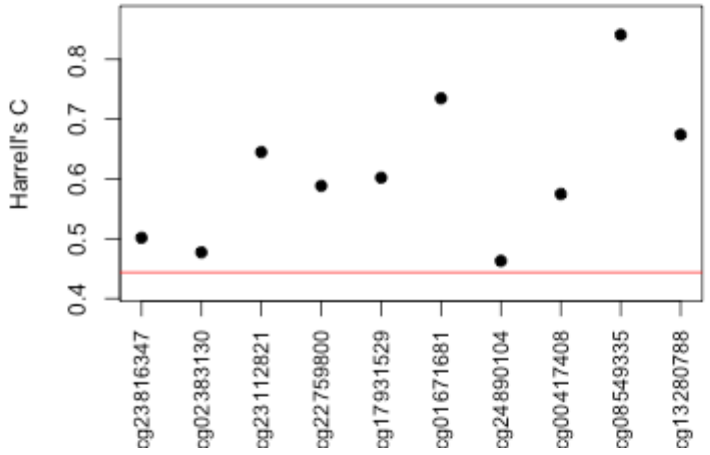

Supplement: Novel epigenetic biomarkers for hematopoietic cancer found in twins [file AO-63-40700-s1.pdf]
